# Mechanical Properties and Constitutive Model Applied to the High-Speed Impact of Aluminum Foam That Considers Its Meso-Structural Parameters

**DOI:** 10.3390/ma14206206

**Published:** 2021-10-19

**Authors:** Qian Guo, Wenbin Li, Wenjin Yao, Xiaoming Wang, Changqiang Huang

**Affiliations:** Ministerial Key Laboratory of ZNDY, Nanjing University of Science and Technology, Nanjing 210094, China; guoqian930327@163.com (Q.G.); njyaowj@163.com (W.Y.); 202xm@163.com (X.W.); cnxahcq@126.com (C.H.)

**Keywords:** aluminum foam, constitutive relationship, cellular structure, numerical simulation, high-speed impact

## Abstract

In this work, quasistatic mechanical compression experiments were used to study the stress–strain relationship of aluminum foam, and the mechanism of the compressive deformation of aluminum foam under quasistatic compression conditions is discussed based on the experimental observations. Since the interactions among cells of the aluminum foam and differences in compressive strength among cells substantially impacted the mechanical properties of the material, the cellular structural parameters, namely the cell size and cell wall thickness, were defined. Along with the mechanism of deformation of a single cell, the influence of structural parameters on the micro failure mechanism and the stress–strain relationship of the aluminum foam material was analyzed. In combination with the factors influencing the mechanical properties of the aluminum foam, a mechanical constitutive model of aluminum foam suitable for multi-density and multi-impact environments that considers cellular structure density was established to predict the complete stress–strain relationship of aluminum foam under a high strain rate. The coupling function of strain rate and temperature in the original model was verified and the parameters were determined by the compression experiments under different strain rates and different temperatures.

## 1. Introduction

Metal foam materials feature a relatively high specific strength and are ideal lightweight structural materials. With the widespread application and rapid development of foam materials, different foam metal production methods will change the material properties and applications, and therefore, the investigation of the mechanical behavior of foam materials has become an important focus for research [1,2,3,4,5,6,7,8]. Aluminum foam and aluminum foam sandwich material can attenuate the amplitude of stress waves during the penetration process because they have a large energy absorption capability that is caused by the plastic yielding and a long, slowly ascending plateau region [9,10,11]. Due to the presence of internal defects in the aluminum foam, the recent experimental data on the performance of metal foams are somewhat varied [12]. Ideally, the stress–strain relationship of the aluminum foam material would exhibit an apparent three-stage relationship, and the mechanical properties of closed-cell aluminum foam would be isotropic [13]. In actual situations, due to the impacts of cell defects and density, the measured values of Young’s modulus and plastic collapse stress of closed-cell aluminum foam are lower than the predicted values of the ideal tetrahedral cell model [14].

The mechanical properties of foam metal are affected by many factors, such as the micro inertia, strain rate sensitivity, restraint, the matrix material’s characteristics, preparation methods, temperature, and microstructure [15,16,17,18,19,20,21]. The strength and hardness of aluminum foam are affected by the distribution of cells. Research conducted by Islam et al. [22,23,24,25] proved that the mechanical properties of aluminum foam are influenced by multiple factors, such as strain rate, impactor shape, and microstructure. Simone et al. [26,27] found that the hardness and strength of honeycomb materials and foam materials are related to cell shape, the shape of cell walls, and the state of the boundary. Liu et al. [28] argued that cell wall strength affects static and dynamic mechanical responses. He et al. [29] proposed a fractal model of aluminum foam materials and hypothesized that the thickness and cell size of cell walls affect the yield stress. Studies by Yang [30] and Chen et al. [31] showed that cell diameter affects the smoothness of the stress–strain curve of aluminum foam. Additionally, for a given density, cell diameter affects the strength of the material. S.K. Nammi [32] established the finite element model of the tetrakaidekahedral repeating unit-cell and found that when the cell size of aluminum foam was smaller, the energy absorption characteristics were stronger, and the peak stress was higher. Hassanli et al. [33] studied the effect of structural design on the mechanical properties of aluminum foam, and from their research, it was shown that the mechanical properties can be improved by the correct modification in pore distribution. Otherwise, the mechanical properties of aluminum foam also have an obvious temperature softening effect. At high temperatures, the plastic deformation of the foam wall will mainly cause holes in the wall, and the mechanical properties will decrease with the increase in temperature. Moreover, the strain rate effect will also change with temperature; therefore, the temperature effect of the material needs to be considered in its constitutive model [34,35].

There are many types of constitutive models of aluminum foam materials, formulated by using the continuous mathematical model to describe the stress–strain relationship of the compressive load response of metal matrix synchronous foams, which is helpful in its application in the numerical simulation [36]. In the existing research on constitutive models of aluminum foam materials, the material density, strain rate effects, and temperature effects are the key research directions [37]. Hu et al. [38] established a one-dimensional viscoplastic hardening model of aluminum foam material under constant temperature and uniaxial compression. Jing Lin et al. proposed a multiparameter nonlinear elasto-plastic constitutive model to describe the typical three-stage features of stress–strain response in aluminum foams. Chen and Lu [39] developed a multiparameter nonlinear elastoplastic phenomenological constitutive model based on the elastoplastic theory, which was able to describe the three-stage characteristics of metal foam. Wang et al. [40] established a quasistatic constitutive model of aluminum foam based on the model of Chen and Lu and demonstrated the accuracy of this model under compression loading. Miller et al. [41] proposed a yield surface function that could be used to describe the plastic behavior of foam metals by using uniaxial compression and tensile experiments, and this model was applied to distinguish the yield responses to tension and compression. Jacques et al. [42] addressed the microscopic scale and considered the impact of the microscale inertia effect to establish an analytical impact model for metal foam. Ding et al. [43] used the Dynamic Rigid-Linear Hardening Plastic-Rigid Unloading model of foam material to determine the dynamic constitutive parameters of aluminum foam. Deshpande and Fleck [44] established the geometrically self-similar and isotropic constitutive model and the differential hardening constitutive model of foam metal through uniaxial compression and hydrostatic compression. A. Reyes [45] evaluated the constitutive model of the aluminum foam developed by Deshpande and Fleck and provided the criteria for the fracturing of foam material. In addition, Liu and Subhash [46] established a multiparameter phenomenological constitutive model of metal foams that considered only a single factor.

The constitutive model of Sherwood and Frost [47] simplified the shape of the stress–strain relationship of foam metal to a single shape function. The basic form of the model is as follows:

(1) $$\sigma =H\left(T\right)G\left(\rho \right)M\left(\varepsilon ,\dot{\varepsilon }\right)f\left(\varepsilon \right)$$

where *H*(*T*) is used to describe the temperature softening, $G\left(\rho \right)$ represents the density effect, $M\left(\varepsilon ,\dot{\varepsilon }\right)$ is considered the strain rate hardening, and *f*(*ε*) is the single shape function, which was used to describe the stress–strain relationship of the foam material.

Hu et al. [48] revised the density strengthening and strain rate strengthening terms; Pengfei Wang [49] established a functional relationship between the strain rate strengthening term and temperature through a high-temperature split Hopkinson pressure bar (SHPB) experiment. Qi et al. [50] proposed a spherical core stratification algorithm for the 3D modeling of aluminum foam. The Sherwood–Frost constitutive framework model was used as the basis for this algorithm, and the influence of relative density was introduced. In addition, based on the Sherwood–Frost model of aluminum foam materials, Gao et al. [51] developed a damage accumulation model for aluminum foam under multiple impacts.

In summary, based on the current research on aluminum foam materials, it has been determined that the cell size and cell wall thickness exert substantial impacts on the mechanical behavior of the material. However, in the constitutive models reported domestically and internationally, relatively little attention has been devoted to these two factors. In this paper, the quasistatic and dynamic compression mechanical behaviors of closed-cell aluminum foam materials are studied, and the mechanism of compressive deformation of aluminum foam is discussed. The influence of cellular structural parameters on the stress–strain relationship is also analyzed. In addition, at its current stage, the constitutive model of aluminum foam considers the material density, strain rate, and temperature. In combination with the dynamic and static mechanical properties of the material, the cell diameter and cell wall thickness are considered, improving the accuracy of the constitutive model.

## 2. Materials and Methods

### 2.1. Materials

In this experiment, closed-cell aluminum foams were provided by Yuan Taida New Material Co., Ltd. (Guangyuan, China). The matrix material was initially 99.7% pure aluminum. Sustained-release casting foaming technology was used in the production process. In this process, the foaming agent (TiH_2_) was added to the melting matrix of pure aluminum material, but the foaming agent’s decomposition was delayed to ensure that it was evenly distributed in the melt. Then, the melt was solidified by casting [52]. After foaming, by using an energy dispersive spectrometer (EDS, Thermo escalab 250Xi, Waltham, MA, USA) and a scanning electron microscope (SEM, Hitachi S4800, Tokyo, Japan), the average content of each component of the aluminum foam material was found—the foam was composed of 83.8% aluminum, 9.2% calcium, 2.8% iron, 2.7% magnesium, and 1.2% titanium. The density of the aluminum foam used in the experiment was 0.23–0.8 g/cm^3^, and the main diameter of the cells in the aluminum foam was 3–6 mm.

**Figure 1:**
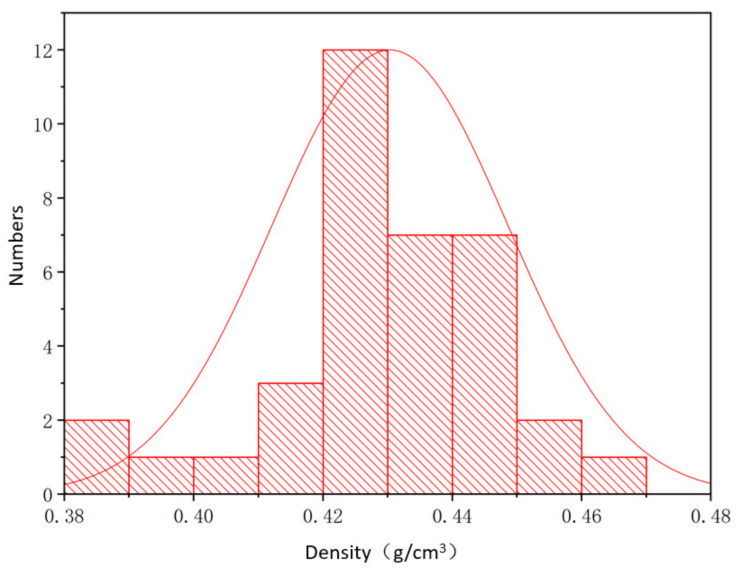
Histogram of the density distribution of 36 specimens (Ø 30 mm × 20 mm).

Aluminum foams produced by the sustained-release cast foaming technology were used. As the thickness of the test piece increased, the density of the test piece measured on the same horizontal plane changed considerably. From the same aluminum foam sample (L × W × T: 200 mm × 200 mm × 30 mm), 36 specimens of dimensions Ø 30 mm × 20 mm were removed, and Figure 1 shows that the density distribution of the aluminum foam material from the same sample basically conformed to a normal distribution. The experimental specimens were processed by wire cutting to avoid the collapse of cell walls and surface distortion.

### 2.2. Experimental Scheme

In this section, a quasistatic compression experiment was performed on closed-cell aluminum foam material. The experiment was carried out using the universal material testing machine (CSS-44300, CIMACH, Changchun, China) at Nanjing University of Science and Technology. Additionally, in this machine, both the deformation sensor and force sensor were used for measurement. The maximum load was 500 KN and the maximum sampling frequency was 50 Hz, the force-measuring accuracy was ±0.5%, and the deformation-measuring accuracy was ±0.5%. The strain rate of the specimen was controlled by controlling the moving speed of the indenter. The data from the testing machine were transferred to a connected computer, real-time pressure values were collected, and the stress of the material was calculated.

(2) $$\sigma =\frac{F_{N}}{A}=\frac{4F_{N}}{\pi D^{2}}$$

(3) $$\varepsilon =\frac{\Delta l}{l_{0}}$$

where σ is the stress of compression, MPa; $F_{N}$ is the reaction force due to the constant-speed crosshead movement-generated displacement, *N*; *A* is the pressure loading area, mm^2^; *D* is the diameter of the specimen, mm; ε is the strain of compression; Δl is the indentation displacement, mm; and $l_{0}$ is the original length of the specimen, mm.

To study the impact of the structural parameters on the stress–strain relationship of aluminum foam, four aluminum foam materials were selected for quasistatic compression experiments. Variation in the materials was mainly determined by their densities. Furthermore, due to the random nature of the density of aluminum foam, when the same group of specimens is selected, the density would not be expected to fluctuate by more than 10%. The samples were grouped by density, as shown in Table 1. Additionally, the velocity of the crosshead was 2 mm/min during the quasi-static compress experiment.

**Figure 2:**
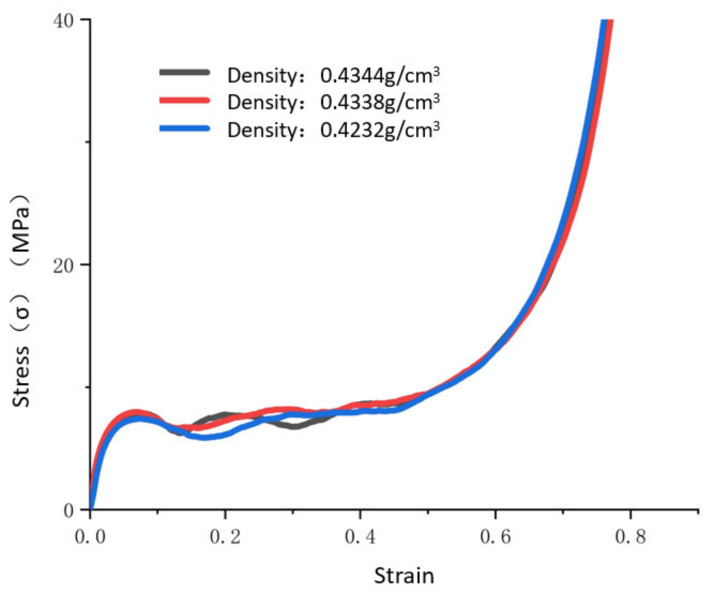
Results of repeated quasistatic experiments on aluminum foam material.

To ensure the accuracy of the experimental results, multiple repeated experiments were carried out in Figure 2. The reason for the fluctuation of the plateau was that the random distribution of the cells of the aluminum foam caused the non-repeatability of internal deformation, leading to slight fluctuations of the yield plateau. Figure 2 shows that the margin of error of the elastic modulus, yield strength, and yield plateau stress is 5%, and the three stress–strain curves of the density scale are approximately coincident.

**Figure 3:**
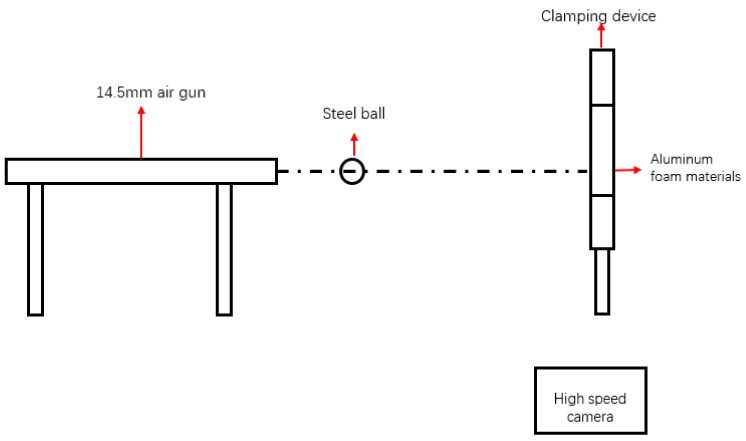
Schematic diagram of the high-speed impact test experimental system.

The high-speed impact test was carried out using an air gun system, with the internal diameter of the barrel being 14.5 mm and 10-millimeter steel balls being used as the projectiles. A Nylon sabot with a diameter of 14.5 mm was used to hold the steel balls during the tests, as shown in Figure 3. The impact target material was a closed-cell aluminum foam material with a density of around 0.423 g/cm^3^. The peripheral regions of the square specimens were fully clamped by 16 bolts, leaving an exposed area of 200 mm × 200 mm. The velocity of the steel spheres before impact with the aluminum foam and the velocity of the steel spheres after penetration of the aluminum foam (residual velocity) were measured using high-speed photography equipment. The results of the high-speed impact test were compared with the results of numerical simulations to verify the modified constitutive model.

## 3. Results and Discussion

### 3.1. The Quasistatic Compressive Deformation Characteristics of the Aluminum Foam Materials under Macroscopic Conditions

**Figure 4:**
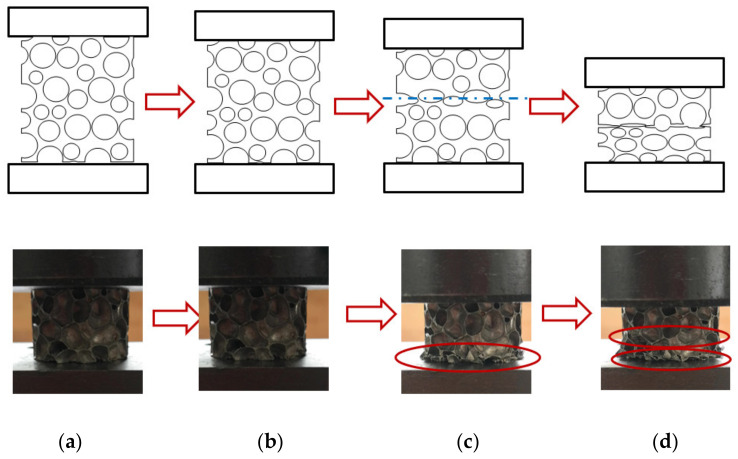
Quasi-static compressive deformation of aluminum foam. Note: The dotted line in the figure is the location of the crushing deformation band. (**a**) Initial state, (**b**) Elastic deformation, (**c**) Plastic yield, (**d**) Plastic collapse.

The actual compressive deformation process of the aluminum foam materials is shown in Figure 4, and a uniform layer-by-layer collapse was observed. In the initial stage of pressure loading, the material exhibited a spatial compression state, the gas in cells was compressed under the load, and cell walls were elastically bent. This stage of the deformation could be restored with pressure unloading, and it was difficult to observe the change in cell shape at this stage with the naked eye, as shown in Figure 4b. The stress–strain relationship at this stage was linear, and the linear slope was the apparent elastic modulus of the aluminum foam material. As the pressure continued to be loaded, the material entered the stage of plastic deformation, and the cells in the specimen at this stage exhibited plastic deformation, including plastic bending, plastic collapse, etc. This stage of the deformation was concentrated in the area of cells with relatively low strength, causing localization of the deformation and forming a crushing deformation band. The other cells remained elastically bent, as shown in Figure 4c. The deformation at this stage was caused by the plastic deformation of the matrix material and was irreversible. In the process of plastic collapse, the plastic deformation of the cells was able to spread to adjacent cells with low strength, causing the cells to be connected. When the cells in the area where the crushing deformation band was located reached maximum density, the stress acting on this layer was released. As the pressure continued to be loaded, a new crushing deformation layer was generated, as shown in Figure 4d. The process repeated itself, and the material attained maximum density. The position of the crushing deformation band was related to the material density, cell distribution, and pressure loading conditions.

### 3.2. Mechanism of Aluminum Foam Deformation at the Mesoscopic Scale

#### 3.2.1. Definition of Structural Parameters

**Figure 5:**
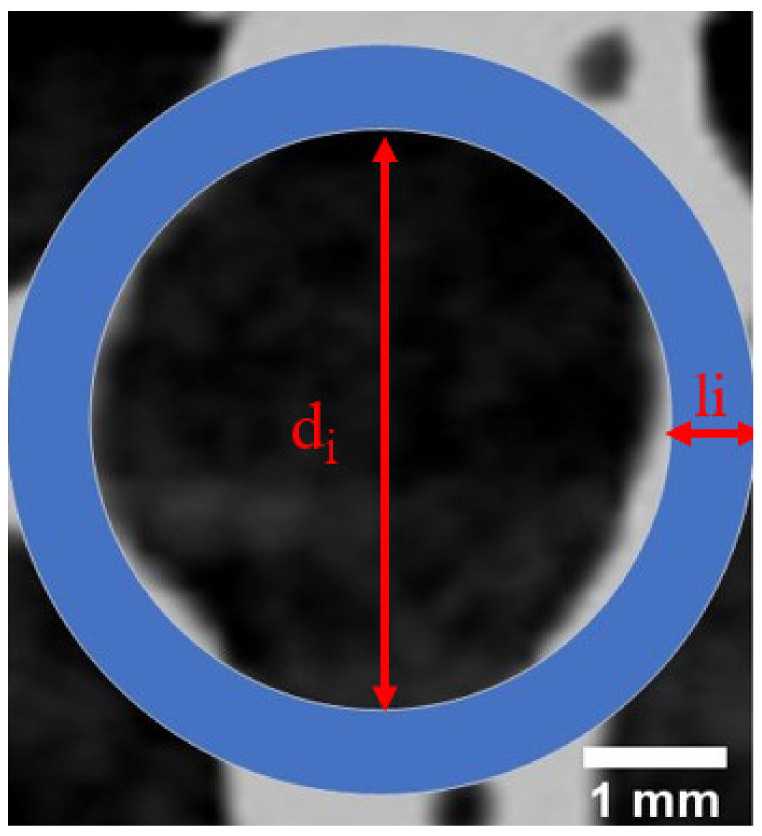
Simplified schematic diagram of the two-dimensional plane of a single cell of aluminum foam material.

Under the condition of quasistatic compression, the mechanism of the macroscopic deformation of aluminum foam is uniform layer-by-layer collapse, but the inhomogeneity of cell distribution can cause the plastic failure mode of the specimen to change with the change in density. To accurately study the mechanism underlying the failure of the mesoscopic compressive deformation of the closed-cell aluminum foam, the influence of cell size and cell wall thickness on the stress–strain relationship needs to be considered. In this paper, the aluminum foam material was simplified as a matrix material with hollow spheres, and cells in a two-dimensional plane were simplified as hollow rings. As shown in Figure 5, the cell size of a single cell is d_i_, and the cell thickness is *l_i_.*

#### 3.2.2. Definitions of Structural Parameters

**Figure 6:**
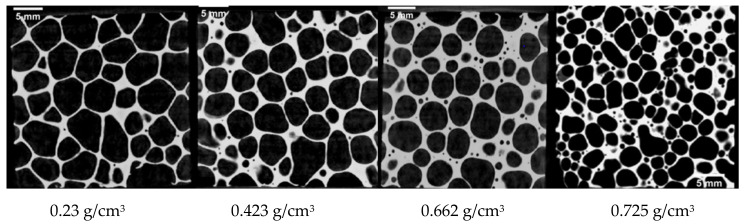
CT scan images of internal holes in the aluminum foam materials with different densities (thickness of 30 mm).

To study the impacts of cell size and cell wall thickness in aluminum foam materials with different densities prepared using the sustained-release foaming method, computerized tomography (CT) scanning was performed on aluminum foam specimens 30 mm in thickness, and the internal structures are shown in Figure 6.

A cell in the aluminum foam material can be simplified as a hollow ring in the two-dimensional plane, as shown in Figure 5, but actual cells exhibited irregular shapes that were approximately circular, as shown in Figure 6. Based on the area of a circle, the cell diameter of a single cell can be defined as a quantity related to the maximum and minimum cell diameters as follows:

(4) $$d_{i}=\sqrt{\frac{d_{imax}^{2}+d_{imin}^{2}}{2}}$$

where $d_{i}$ is the diameter of a single cell, in mm; $d_{imax}$ is the maximum cell diameter of a single cell, in mm; and $d_{imin}$ is the minimum cell diameter of a single cell, in mm. The CT scan images were used for the measurement of the cell size and cell wall thickness of the aluminum foam material. Five planes were selected for measuring cell size and cell wall thickness in two dimensions, and the average values were calculated to obtain the average cell diameter and average cell wall thickness of the aluminum foam material in a two-dimensional plane.

Combined with the CT scanning results, the above method was used to measure the cell parameters, and the statistical method showed that the average cell diameter of 0.423 g/cm^3^ aluminum foam was 3.875 mm, and the average cell wall thickness was 0.588 mm. The average cell diameter of the 0.662 g/cm^3^ aluminum foam was 3.596 mm, and the average cell wall thickness was 0.723 mm, while the average cell diameter of the aluminum foam with a density of 0.23 g/cm^3^ was 4.202 mm, and the average cell wall thickness was 0.58 mm. The average cell diameter of the 0.725 g/cm^3^ aluminum foam was 2.599 mm, and the average cell wall thickness was 0.451 mm. To better study the effect of structural parameters on the quasi-static mechanical behavior of aluminum foam, the cellular structure parameter of aluminum foam in two-dimensional plane is defined as follows:

(5) $$\Omega =\frac{\;\eta }{d}$$

where *d* is the average cell diameter of the aluminum foam material (mm) and *η* is the average cell wall thickness of the aluminum foam material (mm). Finally, grouping was carried out according to the structural parameter Ω as follows: (1) Ω = 0.1380 (aluminum foam with a density of 0.23 g/cm^3^); (2) Ω = 0.1519 (aluminum foam with a density of 0.423 g/cm^3^); (3) Ω = 0.1735 (aluminum foam with a density of 0.725 g/cm^3^); (4) Ω = 0.2011 (aluminum foam with a density of 0.662 g/cm^3^). At different stages of the deformation of aluminum foam, the structural parameters and density should be discussed together.

Table 2 shows the parameter values corresponding to the quasistatic stress–strain curves of aluminum foam materials with different structural parameters. In the quasi-static experiment on aluminum foam, there is a stress-drop stage after the elastic section, and there are fluctuations and strain hardening in the curve due to the uneven distribution of cell pores in the material. In this paper, the measurement and calculation of various parameters followed the following rules: the elastic modulus E is the linear fitting slope when compression produces 0.2% strain, the yield stress is the first peak stress of the stress–strain curve, the yield plateau stress is a stable starting point after the material stress rises, and generally it is the stress average corresponding to 30~40% of the compressive strain. Due to the strain hardening phenomenon of the aluminum foam material in the plateau stage, the densification strain is defined as the strain at the intersection point between the tangent line of the plateau stage and the densification stage tangent, and the starting point of the initial strain of the yield plateau stage of the material is the beginning of the stress recovery—that is, the strain value corresponding to the first trough. The data in Table 2 were obtained by taking the average value of several experimental results, and the ratio range of the measured standard deviation to the average value is less than 10%. Table 2 indicates that the corresponding strain (plastic yield strain) when the material reached the yield stress, the initial strain of the yield plateau stage, and the densification strain of the material increased with increases in cell structural parameters. 

#### 3.2.3. Impacts of Structural Parameters at Each Stage of Compression

**Figure 7:**
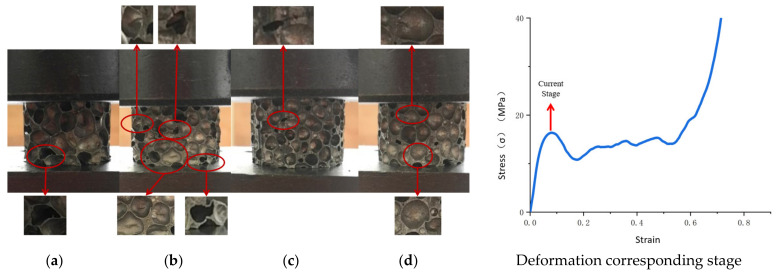
The initial peaks of stress–strain curves of aluminum foam materials with different structural parameters. (**a**) Ω = 0.1380, (**b**) Ω = 0.1519, (**c**) Ω = 0.1735, (**d**) Ω = 0.2011.

With the loading of pressure, the main deformation of a cell at the first peak of the stress–strain curve involved elastic bending and plastic bending Figure 7). At this stage, for aluminum foam with the cell structural parameter Ω = 0.1380, the strength of a single cell was low, and the distribution of cell strength was uniform. Therefore, the plastic bending of the cells mainly occurred at this stage during the compression process. At this stage, the degree of plastic deformation of the specimen was mainly affected by density and cell size. For aluminum foam with Ω = 0.1519, since the compressive stress from the indenter and the compressive stress at the supporting end were not aligned during the pressure loading process, movement occurred between the cells, and wrinkles appeared on the thin walls. For aluminum foam with Ω = 0.1735, the plastic failure occurred earlier due to the stress concentration of the defective portion; for the aluminum foam with Ω = 0.2011, cells were torn due to tensile stress.

**Figure 8:**
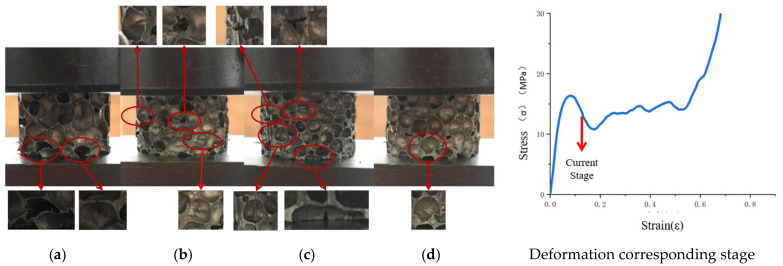
Stress drop of aluminum foam materials with different structural parameters. (**a**) Ω = 0.1380, (**b**) Ω = 0.1519, (**c**) Ω = 0.1735, (**d**) Ω = 0.2011.

Deformation varied substantially among the aluminum foam materials with different cell structural parameters (Figure 8). At this stage, the cell deformation in each aluminum foam specimen was localized, the cells were placed under pressure and underwent plastic deformation, and the plastic bending of the previous stage spread until that portion was crushed and became unstable. An initial deformation band was formed in each specimen in the plastic deformation cell, and as the pressure continued to be loaded, the cells in the deformation band continued to be deformed until they were completely compacted. At this stage, the aluminum foam material with Ω = 0.1380 was still uniformly deformed, and the portion outside the deformation band remained in the original state. The aluminum foam material with Ω = 0.1519 produced broad-area transverse folds in the previous stage. When the plastic deformation reached a certain yield stage, the pressure began to spread to other low-strength areas and produced yield flow, and the folds no longer expanded. With the loading of pressure, in the aluminum foam material with Ω = 0.1519 and the aluminum foam material with Ω = 0.1735, some low-strength single cell walls were subjected to local tensile stresses perpendicular to the direction of compression, resulting in cracks oriented in the direction of the compression. In the aluminum foam material with Ω = 0.2011, the cracks generated in the preceding stage continued to spread to nearby low-strength areas.

**Figure 9:**
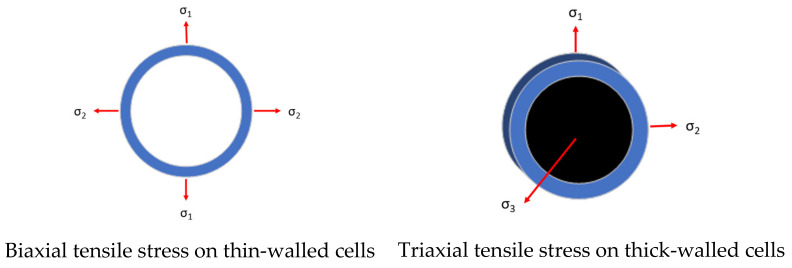
Schematic diagram of stress on cell walls.

At this stage, as the structural parameters increased in value, the cell wall thickness increased, which could cause changes in the cell stress state. The thin-walled cells of the aluminum foam material were mainly subjected to biaxial tensile stress, as shown in Figure 9, which produced cracks in the cell walls in the direction of the compression. As the thickness of a cell wall increased, the stress assumed a triaxial stress state. Therefore, the thick-walled cells of the aluminum foam material were mainly subjected to triaxial tensile stress, as shown in Figure 9; therefore, cracks perpendicular to the compressive direction were generated in the cells.

**Figure 10:**
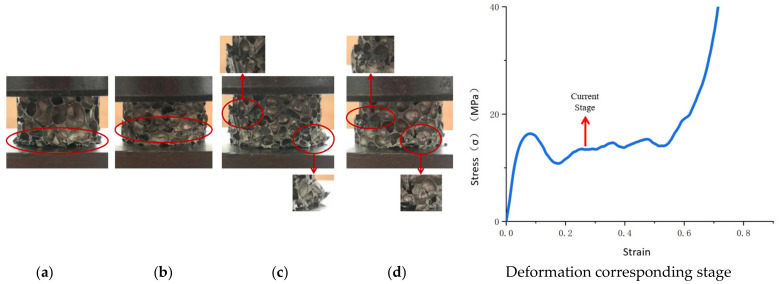
Yield plateau stage of the aluminum foam materials with different structural parameters. (**a**) Ω = 0.1380, (**b**) Ω = 0.1519, (**c**) Ω = 0.1735, (**d**) Ω = 0.2011.

After the aluminum foam material entered the yield plateau stage and the stress stopped rising and stabilized, adjacent cell walls came into contact with each other, the stress of the material increased slowly with increasing strain, and the material entered the uniform collapse stage. In the quasistatic compression experiment, the collapse surface of the aluminum foam varied greatly with changes in structural parameters, as shown in Figure 10. The deformation of the aluminum foam material with Ω = 0.1380 was uniform during the compression process, and there was no breakage during the gradual collapse process. There was no shear deformation between the cells, the specimen was compacted evenly and slowly, and the first collapsed surface was located at the supporting end of the specimen; as the density and values of structural parameters increased, cell cracks in the aluminum foam specimen gradually increased. The collapsed plane was located in the middle of the specimen in the portion where the plastic failure had been fairly concentrated during the first two stages. The collapse process was accompanied by brittle collapse and instability, and finally shear deformation occurred. With increasing values of structural parameters, the material deformation transitioned from a completely elastic-plastic foam to partially brittle foam.

### 3.3. Establishment of a Constitutive Model

Since the constitutive relationship of aluminum foam materials changes with the density of the material and the randomness of the cell distribution, the establishment of a phenomenological macroscopic constitutive model is of great significance to the theoretical analysis of aluminum foam materials. The current research on constitutive models of aluminum foam does not consider the functional relationship between cell size and the cell wall thickness of the material. Assuming that closed-cell aluminum foam is isotropic, we combined the existing constitutive model with an examination of the mechanical properties and microfailure mechanism of aluminum foam and modified the constitutive model by considering the cell’s structural parameters.

Based on the Sherwood–Frost constitutive framework model, the model we developed simplified the shape of the stress–strain relationship of the metal foam to a single shape function, and the basic form is expressed as Equation (1). However, based on the discussion regarding the strain–stress relationship in this paper, the basic form of constitutive model was modified as follows:

(6) $$\sigma =H\left(T\right)G\left(\rho ,\varepsilon ,\Omega \right)M\left(\varepsilon ,\dot{\varepsilon },\;T\right)f\left(\varepsilon \right)$$

where the main modifications of the Sherwood–Frost equation would be to write the part of $G\left(\rho \right)$ as a function of density, strain, and the cell structural parameters in Equation (1).

A single shape function was used to describe the stress–strain relationship of the aluminum foam material, and the shape function was defined as a series related to strain, as follows:

(7) $$f\left(\varepsilon \right)=\overset{n}{\underset{i=1}{\sum }}A_{i}\varepsilon^{i}$$

According to the analysis of the pattern of change in the stress–strain curve of the aluminum foam material, the strain value at each stage of the process is affected by the cell’s structural parameters, and density exerts a greater effect on stress amplitude; therefore, we modified the density-related function to a function depending on both density and cell structural parameters (cell diameter and cell wall thickness) as follows:

(8) $$G\left(\rho ,\varepsilon ,\Omega \right)={\left(\frac{\rho }{\rho_{0}}\right)}^{a}\varepsilon^{B\left(\frac{\Omega_{k}}{\Omega_{0}}-1\right)}$$

where $\Omega_{0}$ = 0.138, $\Omega_{k}$ is the structural parameter; $\rho_{0}$ = 0.237 g/cm^3^, and ρ is the density of the aluminum foam.

The matrix material of pure aluminum foam is a strain rate-sensitive material and shows a temperature effect. In addition, the strain rate effect of the aluminum foam material is affected by temperature, and the stress–strain relationship of the aluminum foam material at high temperatures is affected by a combination of the strain rate strengthening effect and the temperature softening effect. The temperature function could be added to the strain rate strengthening term and combined with the temperature softening term and strain rate strengthening term in the Johnson–Cook (JC) model to form the strain rate and temperature coupling term in the constitutive model of aluminum foam material [53]. By combining the shape function described above with the density function, the constitutive model of aluminum foam material can be obtained as follows:

(9) $$\sigma =\left[1-{\left(\frac{T-T_{room}}{T_{melt}-T_{room}}\right)}^{m}\right]\cdot \left[{\left(\frac{\rho }{\rho_{0}}\right)}^{a}\varepsilon^{B\left(\frac{\Omega_{k}}{\Omega_{0}}-1\right)}\right]\cdot \left[1+C_{0}e^{k\frac{T}{T_{melt}}}\ln\frac{\dot{\varepsilon }}{\dot{\varepsilon_{0}}}\right]\cdot \overset{n}{\underset{i=1}{\sum }}A_{i}\varepsilon^{i}$$

In the equation, $\dot{\varepsilon_{0}}$ = 0.01, and each parameter can be obtained by experimental curve fitting, as shown in Table 3.

**Figure 11:**
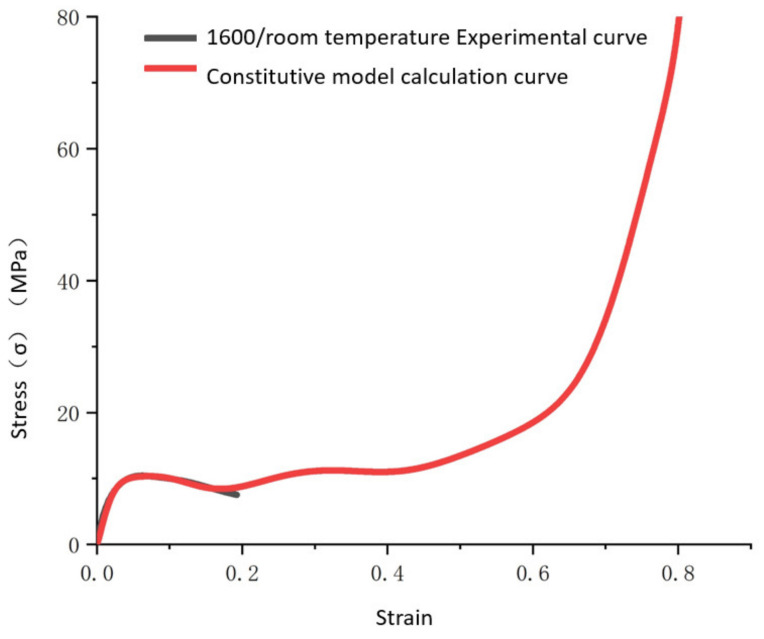
Comparison between fitting curve of 1600/s strain rate constitutive model and experimental c urve at room temperature.

**Figure 12:**
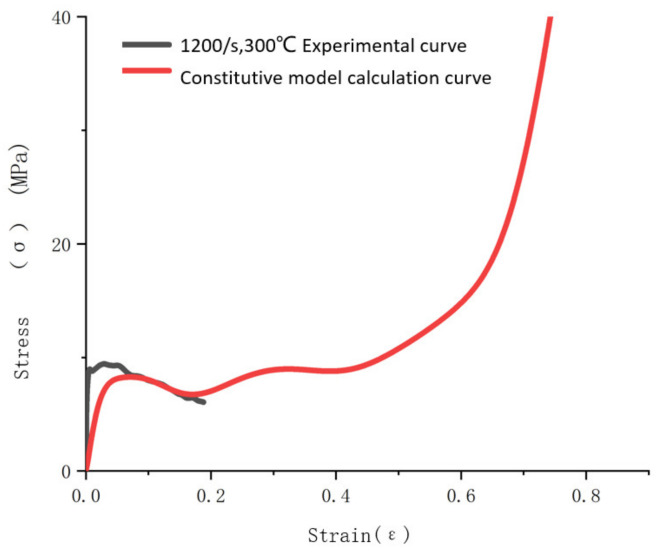
Comparison between fitting curve of 1200/s strain rate constitutive model and experimental curve at 300 °C.

In order to verify the accuracy of the modified constitutive model and the fitting parameters, the dynamic compression experiments of aluminum foam at room temperature and 300 °C were carried out by using the split Hopkinson pressure bar experiment. The stress–strain curves obtained from the experiments were compared with the stress–strain curves calculated using the modified constitutive model, as shown in Figure 11 and Figure 12. It can be seen that the stress–strain curve calculated using the modified constitutive model has high coincidence with the experimental curve at room temperature and high coincidence with the plastic section of the dynamic compression experimental stress–strain curve at a high temperature.

### 3.4. Validation of the Constitutive Model

The constitutive model of aluminum foam material considering the influence of temperature softening, strain rate strengthening, density, and cell structural parameters was used to calculate the theoretical stress–strain curve of the aluminum foam material under a high strain rate. LS-DYNA finite element software and high-speed impact experiments were used to compare the residual velocities of steel spheres with different initial velocities after they impacted the aluminum foam target plate to validate the correctness of the constitutive model.

**Figure 13:**
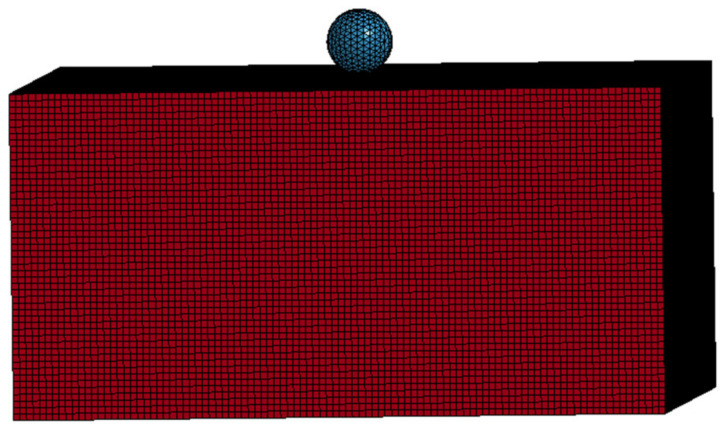
Geometric model of a steel sphere impacting aluminum foam material.

In the numerical simulation study, the No. 63 MAT_CRUSHABLE_FOAM compressible foam model was used as the aluminum foam material. The model can be applied to isotropic foam materials and the strain rate effect of the material is taken into account. In this material model, the constitutive model of the material is not defined, and the constitutive relationship of the material is described by drawing the stress–strain curve of the material during the numerical simulation. Due to the difficulty in obtaining the complete stress–strain curve of aluminum foam under a high strain rate, the stress–strain curve under quasi-static stress is usually used for numerical simulation. In the simulation of high-speed impact in this paper, the modified constitutive model was used to calculate the complete stress–strain curve under a high strain rate, and the curve was attached to this material model. The steel sphere was defined as a rigid body, and the No. 20 material model MAT_RIGID was used for calculation. A geometric model of a high-speed steel sphere impacting the aluminum foam target is shown in Figure 13. The uniform mesh is adopted in the whole model, and the total amount of mesh of the aluminum foam is 500,000, and the number of steel balls is 3753. The target plate has no constraint in the moving direction of the steel ball, and the other four degrees of freedom are fully constrained. Considering the large deformation of the soft material, the CONTACT_INTERIOR keyword was added to the aluminum foam to avoid a negative volume of soft materials in the compression and shear mixing mode.

**Figure 14:**
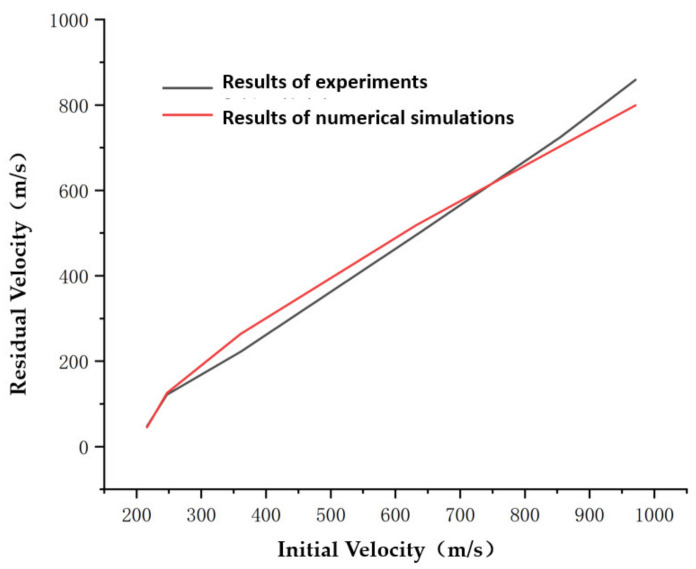
Results of experiments and numerical simulations for steel spheres with different initial velocities impacting aluminum foam materials.

The model was used to numerically simulate the process of steel spheres with different initial velocities impacting aluminum foam. The resulting residual velocities of the steel spheres obtained in the experiment were compared with the results obtained from the numerical simulation, as shown in Figure 14.

The residual velocity error *φ* of the numerical simulation was calculated as follows:

(10) $$\varphi =\frac{V_{rs}-V_{re}}{V_{re}}$$

where $V_{rs}$ is the residual velocity obtained from numerical simulation and $V_{re}$ is the residual velocity obtained from the experiment, in m/s.

Figure 14 and Table 4 show that the constitutive relationship of aluminum foam established in this paper was able to keep the numerical simulation error within ±10%, and the numerical simulations were consistent with the experimental results, which validated the effectiveness of the constitutive model of the aluminum foam material.

**Figure 15:**
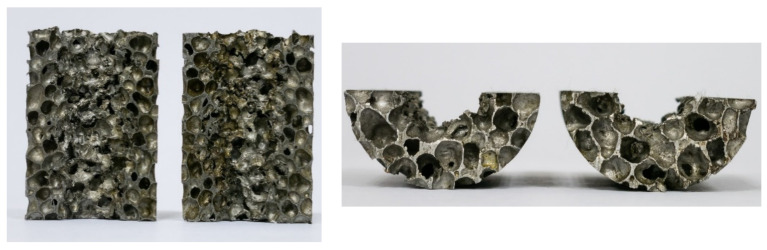
Cutting image of trajectories of steel balls penetrating aluminum foam.

In the high-speed impact test, the steel ball compacted the aluminum foam in the area below the contact surface, and the compacted area and the surrounding cellular structure were torn under the shear force. During the penetration process, due to the tearing effect of the steel ball on the aluminum foam, the aluminum foam has brittle failure, produces debris, and forms a collision zone with the steel ball, and continues to propagate in the aluminum foam. When the steel ball moves to the back of the aluminum foam material, it will tear the back of the foam plate under the action of tensile stress and form a breach. The steel ball and the fragments of the aluminum foam leave the target plate, and the trajectory of the steel ball in the aluminum foam material is shown in Figure 15. The plastic deformation caused by high-velocity impact on the aluminum foam is mainly concentrated near the trajectory line of the steel ball. The aluminum foam only partially fails, and the rest remains intact.

**Figure 16:**
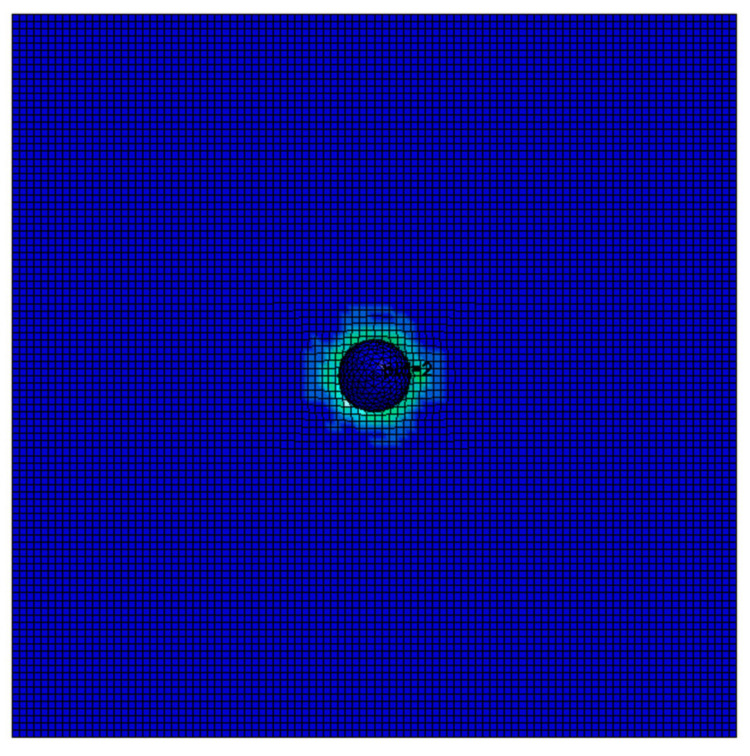
Von Mises stress of numerical simulation model of steel ball impacting aluminum foam.

**Figure 17:**
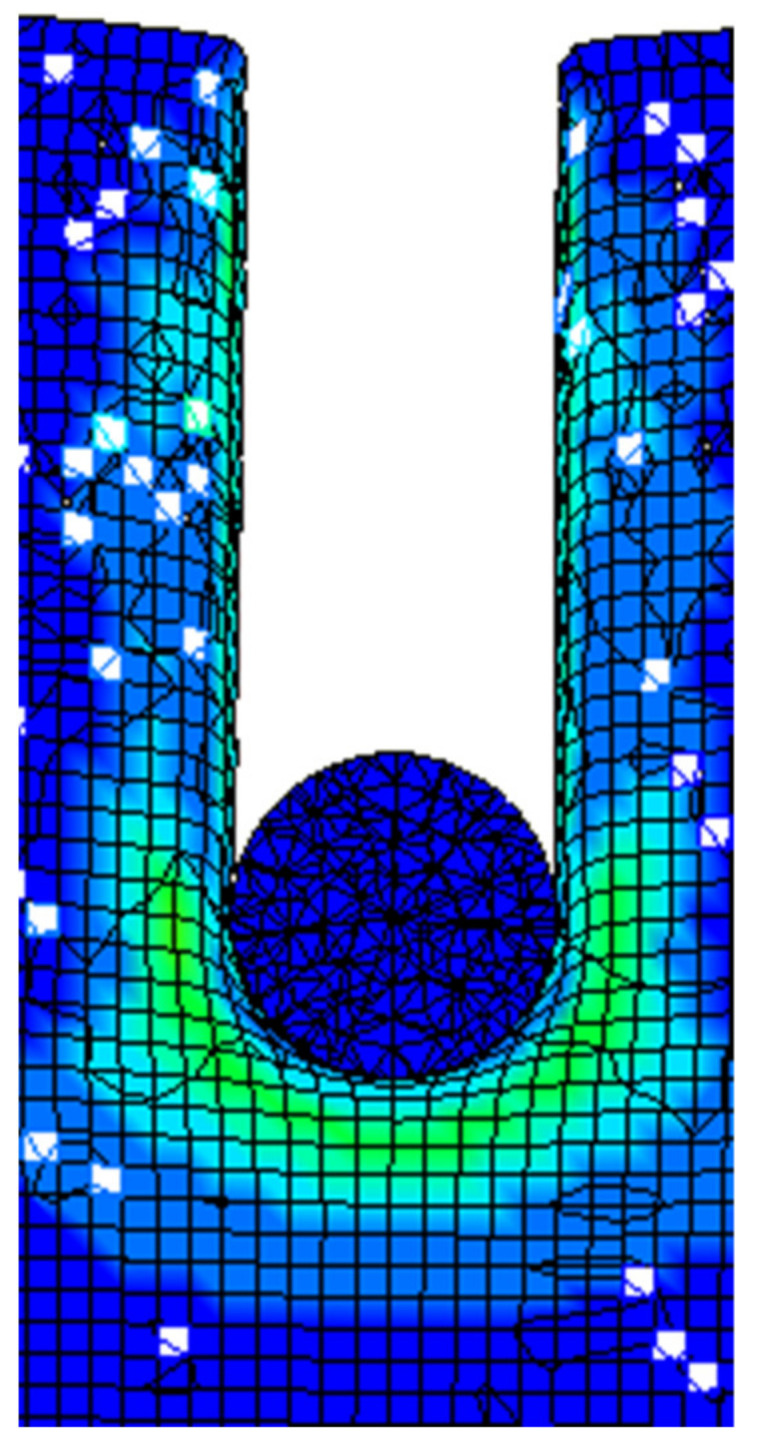
Cutting image of steel ball impacting aluminum foam.

**Figure 18:**
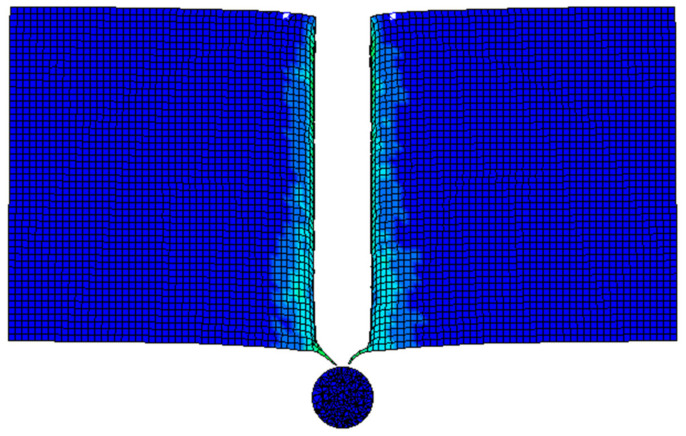
Plate sectional stress of steel ball impacting aluminum foam.

The stress of Von Mises of aluminum foam shows that stress is mainly concentrated near the steel ball trajectory, as shown in Figure 16. It can be seen from the cutting off model that when the steel ball moves into the aluminum foam material, it will cause the mesh extrusion deformation below the contact area, and the rest of the mesh will maintain the initial state, as shown in Figure 17. It can also be observed that the stress in the aluminum foam material is mainly concentrated near the steel ball trajectory, and the area far from the steel ball will not be affected by the force. This is due to the good energy absorption effect of the aluminum foam material, which causes the damage to be concentrated in the penetration area and plays a protective role in other regions, as shown in Figure 18.

**Figure 19:**
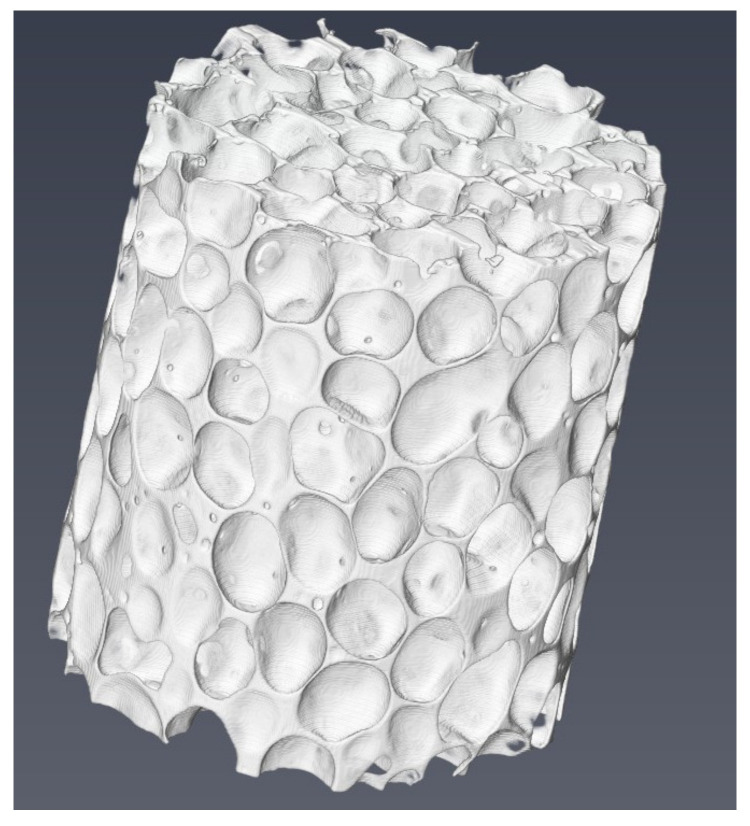
Three-dimensional modeling of aluminum foam based on CT scanning tomography technology.

**Figure 20:**
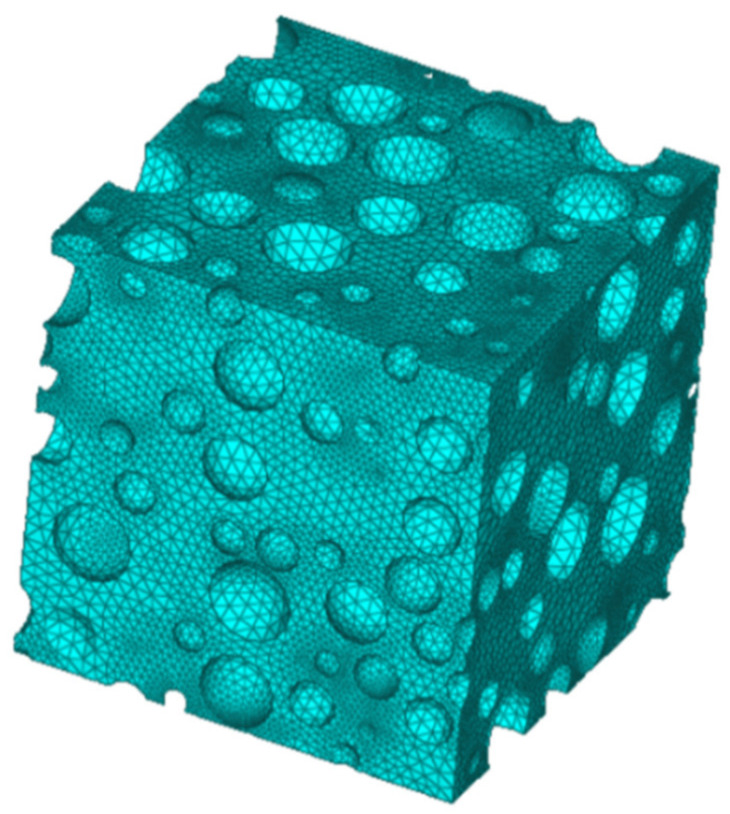
Three-dimensional randomly distributed spherical cell model.

At the same time, we established the discrete model based on CT scanning tomography technology and the mesoscopic finite element model of aluminum foam based on the random model established by irregularity, as shown in Figure 19 and Figure 20. The constitutive model of aluminum foam is selected by the Johnson–Cook constitutive model in the mesoscopic finite element model. When the initial velocity of the steel ball is 287 m/s, the residual velocity of the mesoscopic finite element model is 178 m/s, and the calculation error with all finite models can be controlled within 10%. Due to the existence of cells in the mesoscopic finite element model, the meso-model can better reflect the interaction between the steel ball and material cells, but its computational efficiency is lower than that of the homogenized finite element model. The constitutive model of the aluminum foam used in this paper is accurate in describing the yield plateau of materials when subjected to a high-speed impact and is a reasonable simplification method in engineering.

## 4. Conclusions

(1) In this paper, aluminum foam was modeled as a hollow ring in a two-dimensional plane. The cell structural parameters of the aluminum foam material in the two-dimensional plane were defined. The quasistatic compressive deformation process of the aluminum foam was divided into five stages, namely, the initial state, elastic stage, “stress drop” stage, yield plateau stage, and densification stage. Meanwhile, the microscopic deformation mechanisms of the cells of aluminum foam materials with different cellular structural parameters at different stages were analyzed sequentially.
(2) The structural parameters mainly affected the magnitude of strain of the aluminum foam in each stage and the width of the plateau stage of the aluminum foam. In terms of the microscopic deformation of the material, with increasing cell wall thickness, the stress state of a cell changed from biaxial tensile stress to triaxial tensile stress. The tensile stress state affected the changes in the direction of cell cracks of the aluminum foam.
(3) Based on the Sherwood–Frost constitutive framework model, a constitutive model was established, which aimed to simulate the shape function, the coupling function of density and cellular structural parameters, and the coupling function of strain rate and temperature. The influence of density and cellular structure was considered when establishing the model, and the results were obtained by curve fitting. High-speed impact tests and numerical simulations were used to validate the accuracy of the constitutive model in practical applications.

## Figures and Tables

**Table 1:** Grouping of samples in the experimental study of stress–strain relationships in different aluminum foam materials.

No.	Pressure State	Density Range
1	Quasi-static experiment	0.23–0.25 g/cm^3^
2		0.41–0.45 g/cm^3^
3		0.62–0.68 g/cm^3^
4		0.73–0.79 g/cm^3^

**Table 2:** Quasistatic stress–strain curve parameters of aluminum foam materials with different cell structural parameters.

Structural Parameter Ω	Density (g/cm^3^)	Plastic Yield Strain	Elastic Modulus (MPa)	Yield Stress (MPa)	Initial Strain of Yield Plateau Stage	Yield Plateau Stress (MPa)	Densification Strain
0.138	0.237	0.0351	78.57	2.043	0.0914	2.07	0.738
0.1519	0.423	0.0654	252.78	7.41	0.1695	7.89	0.687
0.1735	0.725	0.0685	491.47	19.779	0.1727	14.5	0.673
0.2011	0.662	0.0787	401.11	16.356	0.1764	14.23	0.637

**Table 3:** Fitting parameters of the modified constitutive model.

Parameter	A_1_	A_2_	A_3_	A_4_	A_5_	A_6_
Value	126.71	−3017.97	32,720.82	−191,926.73	669,897.0082	−1,455,540
Parameter	A_7_	A_8_	A_9_	A_10_	a	B
Value	1,986,420	−1,653,940	766,307.24	−150,868.865	1.82	0.05
Parameter	m	k	$C_{0}$			
Value	1.08	1.754	0.0266			

**Table 4:** Differences between numerical simulation results and experimental results for steel spheres with different initial velocities impacting aluminum foam materials.

Initial Velocities (m/s)	Residual Velocity of Experiments (m/s)	Residual Velocity of Numerical Simulation (m/s)	Result Error (%)
216	48	45.2	−5.833
247	122	125.3	2.70
632	495	518.3	4.71
855	725	704	−2.9
971	859	799	−6.9

## Data Availability

Data available on request due to restrictions privacy or ethical. The data presented in this study are available on request from the corresponding author.
